# The role of the Mediterranean diet in the treatment of cognitive dysfunction in patients with type 2 diabetes mellitus

**DOI:** 10.3389/fnut.2025.1654684

**Published:** 2025-08-14

**Authors:** Chunyan Zheng, Qinqin Zhang, Fengli Liu, Guihong Qiu

**Affiliations:** ^1^Cadre Health Care Office, Central Hospital of Zibo, Zibo, Shandong, China; ^2^Department of Otolaryngology, Central Hospital of Zibo, Zibo, Shandong, China; ^3^Department of Internal Medicine, Zibo Maternal and Child Health Hospital, Zibo, Shandong, China

**Keywords:** Mediterranean diet, type 2 diabetes mellitus, cognitive dysfunction, insulin resistance, chronic hyperglycemia, dyslipidemia

## 1 Introduction

The global metabolic disorder, type 2 diabetes mellitus (T2DM), significantly increases the risk of cognitive dysfunction, including Alzheimer's disease (AD) and mild cognitive impairment (MCI). Evidence shows that there is the same pathophysiological mechanism between T2DM and neurodegeneration (1–4). The Mediterranean diet (MedDiet)—a dietary pattern rich in fruits, vegetables, whole grains, nuts, fish, and olive oil, with low saturated fat intake—may improve cognitive dysfunction of T2DM. However, challenges such as the lack of a unified MedDiet definition and compliance standards make translational research difficult to operate. This opinion article summarizes the current understanding of the pathological mechanisms that link T2DM with cognitive dysfunction and evaluates the protective effects of the MedDiet. The aim is to inform therapeutic strategies that could delay or prevent cognitive decline associated with T2DM. Additionally, it analyzes the potential challenges related to implementing the MedDiet in the context of T2DM-associated cognitive impairment.

## 2 Pathophysiological links between type 2 diabetes mellitus and cognitive dysfunction

### 2.1 Insulin resistance and brain insulin signaling

Peripheral insulin resistance (IR) in T2DM disrupts central insulin signaling through various mechanisms, leading to neurodegeneration. Hyperinsulinemia, a characteristic feature of T2DM, reduces insulin passing through the blood–brain barrier (BBB) by downregulating insulin receptors (5). This process diminishes insulin availability in critical brain regions, such as the hippocampus and cortex (6). Decreased insulin signaling impedes the phosphatidylinositol 3-kinase/protein kinase B (PI3K/AKT) pathway, which is crucial for neuronal survival, synaptic plasticity, and glucose metabolism (7). Inhibition of PI3K/AKT activity triggers the activation of glycogen synthase kinase-3β (GSK-3β), resulting in tau hyperphosphorylation and the formation of neurofibrillary tangles—a pathological marker of AD (8, 9). Meanwhile, the decrease in insulin-degrading enzyme (IDE) activity due to IR can diminish amyloid-β (Aβ) clearance, accelerating the deposition of Aβ plaques (10). Aβ aggregates further exacerbate insulin resistance by binding to neuronal insulin receptors and activating pro-inflammatory pathways, forming a vicious cycle.

Chronic peripheral insulin resistance can also induce neuroinflammation by causing systemic metabolic disorders. Elevated levels of free fatty acids and cytokines, such as TNF-α and IL-6, activate microglia and astrocytes, causing oxidative stress and permanent synaptic damage (11). Inflammatory mediators disrupt the integrity of the BBB, allowing peripheral inflammatory molecules to penetrate the brain and exacerbate neuronal inflammation. In addition, advanced glycation end products (AGEs) caused by hyperglycemia interact with AGE receptors on neurons, triggering NF-κB activation and further enhancing the production of Aβ and tau pathology (12–14). Neuroimaging studies have shown that the utilization rate of glucose in the brain of patients with T2DM is reduced and the hippocampus is atrophied (15), which correlates with impairments in memory and executive function. These findings highlight that peripheral insulin resistance plays an important role in the dysfunction of brain insulin signaling and AD-like changes in T2DM.

### 2.2 Dyslipidemia and lipid metabolism dysregulation

Dyslipidemia, also known as lipid metabolism disorders, involves imbalances in cholesterol, triglycerides, and other lipid components. These imbalances are closely linked to cardiovascular diseases, obesity, and metabolic syndrome. The process of lipid metabolism changes with age (16).

The key subtype of dyslipidemia, dysregulation of cholesterol metabolism, and the synergistic effect of the ApoE4 allele, the main genetic risk factor of AD, exacerbate AD-related pathological changes. ApoE4 reduces the efficiency of cholesterol transport in the brain, decreasing the clearance of Aβ peptides, while dysregulated cholesterol metabolism promotes the abnormal processing of the amyloid precursor protein (APP), increasing Aβ production (17, 18). The above dual mechanisms amplify Aβ deposition, while cholesterol imbalance activates kinases such as GSK-3β, driving tau hyperphosphorylation and the formation of neurofibrillary tangles (19). ApoE4 accelerates neuronal damage and further exacerbates tau pathology by disrupting microtubule stability and reducing tau degradation. In patients with T2DM, chronic hyperglycemia and IR will destroy brain lipid homeostasis and aggravate the imbalance of cholesterol metabolism. The metabolic stress associated with T2DM impairs ApoE-mediated clearance of Aβ, which synergizes with the intrinsic clearance deficiency of ApoE4 to accelerate the accumulation of Aβ. Meanwhile, hyperglycemia and abnormal lipid metabolism enhance tau phosphorylation, while ApoE4 enhances tau pathology by altering intracellular signaling. These interactions collectively elevate the dementia risk in T2DM patients carrying ApoE4 far beyond that of individuals with either condition alone.

### 2.3 Vascular injury and cerebral hypoperfusion

Vascular injury and cerebral hypoperfusion are critical contributors to cognitive dysfunction in patients with T2DM, with chronic hyperglycemia playing a central role in these pathological processes. Prolonged hyperglycemia directly damages vascular endothelial cells, the protective barrier between blood and vessel walls, by activating the TLR4 signaling pathway. This activation promotes the release of pro-inflammatory cytokines (e.g., IL-6 and TNF-α) and triggers oxidative stress through excessive production of reactive oxygen species (ROS) (20, 21). Endothelial damage disrupts the balance between the anticoagulant and procoagulant functions of endothelial cells, leading to platelet aggregation, lipid deposition, and the formation of atherosclerotic plaques. Consequently, vascular stenosis and stiffening occur, which reduces cerebral blood flow and impairs brain perfusion.

Cerebral blood insufficiency reduces the supply of nutrients and oxygen to neurons, particularly affecting microvascular networks that are critical for maintaining cognitive function (22). Microvascular complications, including diabetic retinopathy (a marker of cerebral microvascular injury), are strongly associated with more severe cognitive decline (23). Research has shown that extensive microvascular damage can affect synaptic plasticity and neuronal metabolism (24). Additionally, chronic hyperglycemia-induced endothelial dysfunction weakens the BBB, increasing the risk of cerebral edema, hemorrhage, and the accumulation of neurotoxic substances such as β-amyloid, which further exacerbates neuronal damage.

Beyond microvascular injury, large-vessel atherosclerosis, such as carotid artery stenosis, significantly impacts cognitive networks by altering cerebral hemodynamics. Studies have shown that the severity of carotid stenosis in patients with T2DM correlates with the extent of cognitive impairment, with severe stenosis doubling the risk of cognitive decline (25). Systemic atherosclerosis also accelerates the progression of cognitive deficits, as a decrease in blood flow in key brain areas, such as the left wedge and superior occipital gyrus, will damage visual processing, memory, and attention, which are domains critical for maintaining cognitive function. Asymptomatic cerebral infarction and microbleeds can further impair cognitive networks, particularly in the domains of attention and processing speed (26). Collectively, these vascular pathologies create a vicious cycle: hyperglycemia leads to endothelial injury and hypoperfusion, which in turn exacerbates metabolic dysregulation and accelerates cognitive decline in patients with T2DM.

## 3 Mechanisms of MedDiet in T2DM-related cognitive dysfunction

The MedDiet refers to the lifestyle of residents in the Mediterranean region, rather than a simple, strict dietary plan. Its core concepts encompass consuming more legumes, vegetables, fruits, nuts, whole grains, spices, and fish (with olive oil as a key source of fat); consuming fish or other seafood at least twice a week; moderately consuming dairy products and eggs; eating less red meat and desserts; and drinking a small amount of wine. It also encourages people to interact with each other and to savor healthy, fresh foods. This dietary pattern is low in saturated fat, accounting for ~10% of energy intake (27, 28), and is rich in various micronutrient functional components, including vitamins, carotenoids, unsaturated fatty acids, and diverse bioactive plant phenolic compounds with antioxidant and anti-inflammatory effects. These plant-derived phenolic compounds may regulate insulin action and metabolism in insulin-sensitive tissues, exerting effects that prevent or treat insulin resistance and its related diseases (29). Initially, Ancel Keys, an expert in animal physiology and biology, discovered through experiments that Italians had a lower prevalence of heart disease and coronary artery disease (30, 31). However, his views were challenged by other scholars. In response, he conducted the renowned “Seven Countries Study,” which confirmed that the Mediterranean dietary pattern was associated with a lower risk of cardiovascular events (32, 33). It is hypothesized that such effects may contribute to a reduction in the risk of dementia (34–36). Building on his research, the Mediterranean dietary pattern has gradually gained widespread recognition.

**Figure 1:**
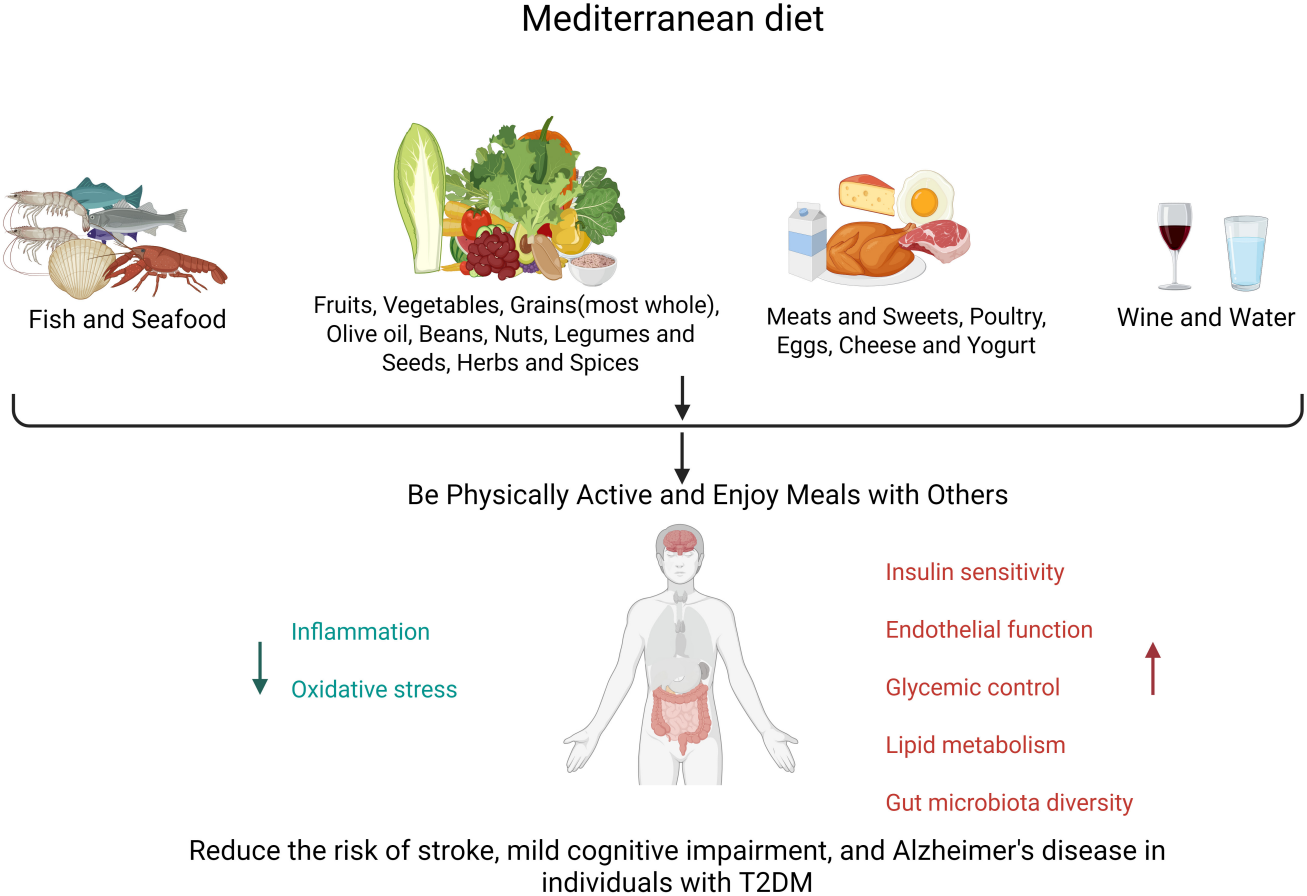
Composition of the MedDiet and its role in cognitive impairment caused by T2DM.

The MedDiet has been proven to better ameliorate IR in obese individuals, with its induced reductions in insulin levels and other IR markers—such as the homeostatic model assessment (HOMA) index—being early and sustained (37, 38). A meta-analysis encompassing several randomized trials, including the large-scale PREDIMED trial (34), revealed that the MedDiet, when compared to low-fat diets, decreased the risk of stroke (HR = 0.60, 95% CI = 0.45–0.80). Another observational study reported lower incidence rates of Parkinson's and AD among individuals adhering to the MedDiet (39). An observational study that utilized dietary questionnaires to evaluate and quantify dietary adherence among various population groups found that individuals with the highest adherence to the MedDiet had lower incidence rates of MCI and AD, as well as slower rates of cognitive decline (Figure 1), compared to those with poor adherence (40–43). A 4.2-year observational study in Israel found that strict adherence to a MedDiet in patients with type 2 diabetes was associated with slower cognitive decline (44). To further explore the key components of the Mediterranean dietary pattern, scholars conducted a follow-up of ~250,000 participants for 11.4 years and found that consuming 2–4 servings of fish per week and 1–2 servings of fruits per day were associated with a reduced risk of dementia (45).

The primary role of the MedDiet lies in its positive effects on glucose metabolism, including improvements in IR, enhancements in insulin clearance, and the strengthening of β-cell function (46). It counteracts cognitive decline through the antioxidant and anti-inflammatory properties of polyphenols (found in olive oil and berries) and omega-3 fatty acids (present in fish) (47–49). It stabilizes glucose levels, improves lipid profiles (reducing LDL-C while increasing HDL-C) (28), enhances endothelial function for cerebrovascular health (50), and modulates the gut–brain axis through its high fiber content, which fosters beneficial microbiota (51, 52). Cross-sectional studies indicate that high adherence is associated with improved verbal memory in diabetes patients (*P* = 0.043), whereas the PREDIMED trial confirmed a 30% reduction in diabetes incidence and a slowdown in cognitive decline among high-risk groups (53). When combined with aerobic exercise, it synergistically enhances glycemic control, lipid metabolism, and gut microbiota diversity. Metformin, a GLP-1 receptor agonist, and other hypoglycemic drugs may reduce cognitive dysfunction in T2DM, a finding that warrants further study.

However, the MedDiet currently lacks a standardized definition, adherence criteria, and scoring system. This significantly hampers comparisons between studies and impedes the translation of scientific research into practical recommendations for the general public (54). On the other side of the shield, despite the well-established benefits of the MedDiet, researchers acknowledge that uncertainties persist regarding its implementation in non-Mediterranean regions. Adherence to the MedDiet poses a common challenge, as it is often perceived as difficult to maintain in non-Mediterranean areas (55).

## 4 Conclusion

T2DM and cognitive dysfunction share common pathophysiological mechanisms. MedDiet's anti-inflammatory, antioxidant, and metabolic regulatory effects provide a promising intervention for patients with cognitive impairment of T2DM. By improving insulin sensitivity, stabilizing glucose levels, optimizing lipid metabolism, and improving cerebrovascular function, MedDiet has been proven to reduce the risk of stroke, MCI, and AD in T2DM patients. Observational studies, such as PREDIMED, have demonstrated their role in slowing cognitive decline, particularly when combined with lifestyle changes including exercise.

There are still challenges regarding MedDiet, including standardization of MedDiet and long-term adherence strategies, especially in non-Mediterranean regions. Research on MedDiet bioactive ingredients for specific pathologies may become the future research direction, and dietary interventions will be included in the personalized management of type 2 diabetes to maximize cognitive protection. In general, these efforts may bridge the gap between basic research and clinical practice and ultimately reduce the burden of cognitive dysfunction associated with T2DM.
